# Haim-Munk syndrome

**DOI:** 10.4103/0972-124X.75919

**Published:** 2010

**Authors:** Priyanka Pahwa, Arundeep K. Lamba, Farrukh Faraz, Shruti Tandon

**Affiliations:** *Department of Periodontics and Oral Implantology, Maulana Azad Institute of Dental Sciences, University of Delhi, Government of National Capital Territory of Delhi, India*

**Keywords:** Early onset periodontitis, Haim-Munk syndrome, palmoplantar keratosis, Papillon-Lefèvre syndrome

## Abstract

Haim-Munk syndrome is an extremely rare autosomal recessive disorder of keratinization characterized clinically by palmoplantar hyperkeratosis, severe early onset periodontitis, onychogryphosis, pes planus, arachnodactyly, and acro-osteolysis. Recently, germline mutations in the lysosomal protease cathepsin C gene have been identified as the underlying genetic defect in Haim-Munk syndrome and in the clinically related disorders, such as Papillon-Lefèvre syndrome and prepubertal periodontitis. The periodontal disease associated with these syndromes is particularly aggressive and unresponsive to traditional periodontal therapies. As a result, most patients become edentulous by 15 years of age. This case report describes a patient with the cardinal features of Haim-Munk syndrome.

## INTRODUCTION

Palmoplantar keratoderma (PPK) is a heterogeneous condition characterized by hyperkeratosis and erythema of the soles of feet and palms of hands. Papillon-Lefèvre syndrome (PLS)[1,2] and Haim-Munk syndrome (HMS) are rare autosomal recessive type IV PPK characterized by the presence of early onset periodontal disease.[3] Patients with HMS also show hypertrophy and curving of nails (onychogryphosis), flat foot (pes planus), extreme length and slenderness of fingers and toes (arachnodactyly), and osteolysis involving the distal phalanges of fingers and toes (acro-osteolysis).

In this case report, a 12-year-old girl and her sibling are described who presented with the cardinal features of HMS.

## CASE REPORT

### Proband

**Figure 1:**
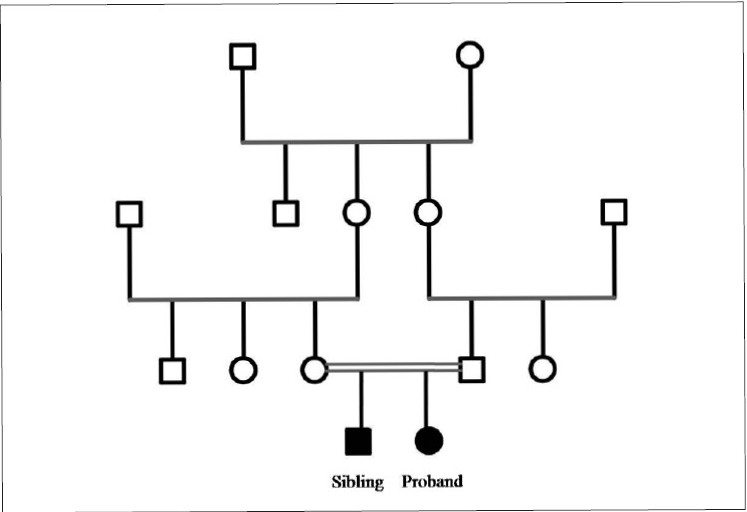
Pedigree chart of the consanguineous family (□: unaffected male; ○: unaffected female; ○=□ consanguineous marriage; ■: affected male; ●: affected female)

A 12-year-old girl reported with the chief complaint of bleeding gums and mobility of teeth. She had normal eruption of deciduous teeth, but early shedding starting at three years of age with complete shedding by the age of six years. She also suffered from dry and thickened skin with recurrent skin infections. Family history revealed that she was born of consanguineous marriage [Figure 1] and there was presence of similar features in her younger brother.

**Figure 2:**
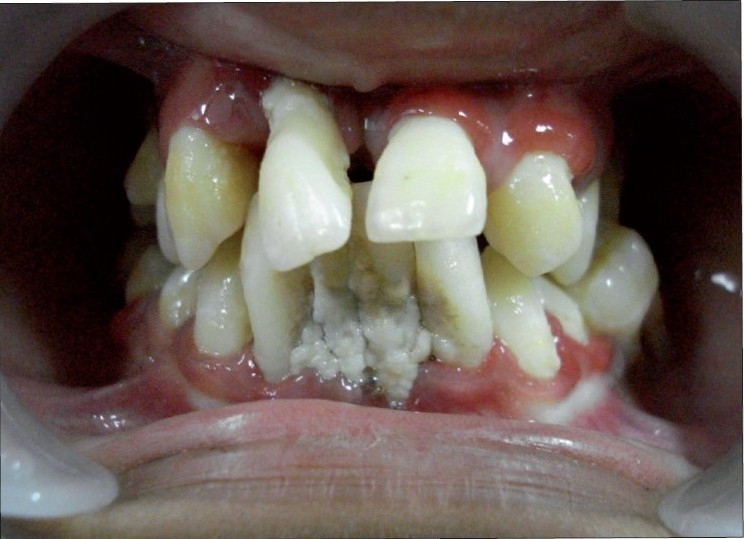
Intraoral appearance. Note the abundance of plaque accumulation and gingival inflammation

**Figure 3:**
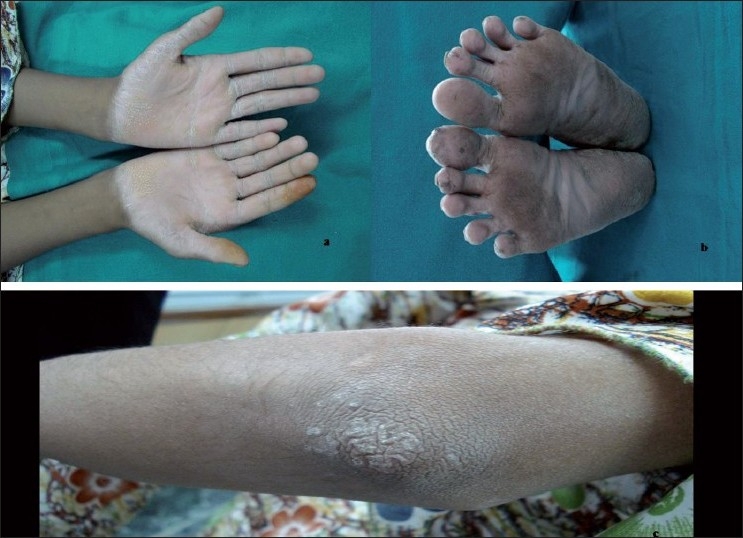
Extraoral appearance. Note severe hyperkeratosis of (a) palms, (b) soles and (c) elbow

**Figure 4:**
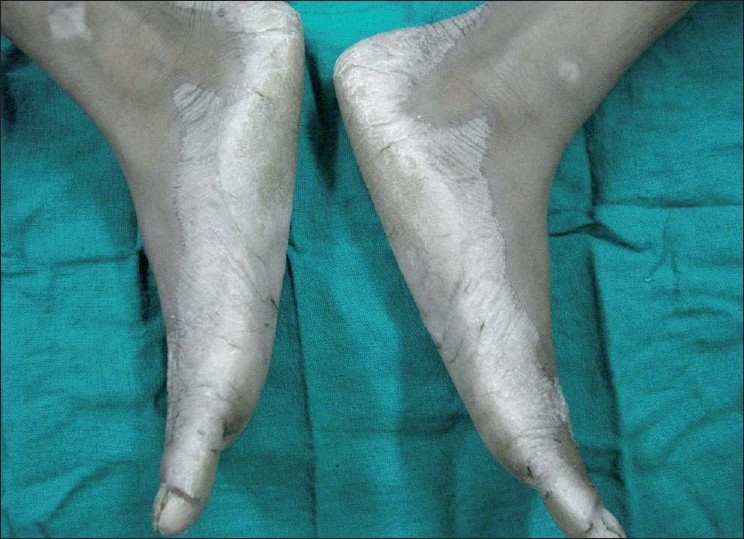
Loss of the medial longitudinal arches of the feet was evident with bilateral pes planus

On intraoral examination, marked drifting of teeth was noticed. Her oral hygiene was extremely poor, with an abundance of plaque accumulation [Figure 2]. The gingiva was red, soft and edematous with profuse bleeding on probing. General examination showed symmetrical, well demarcated, keratotic and confluent plaques affecting the skin of palms, soles and elbows [Figure 3]. The lesions on the soles extended onto the lateral malleoli and the Achilles’ tendons. The skin was dry and rough to touch. The nails showed transverse grooving and slight pitting. The patient had a history of recurrent skin infections. Loss of the medial longitudinal arches of the feet was evident with bilateral pes planus [Figure 4].

### Investigations

**Figure 5:**
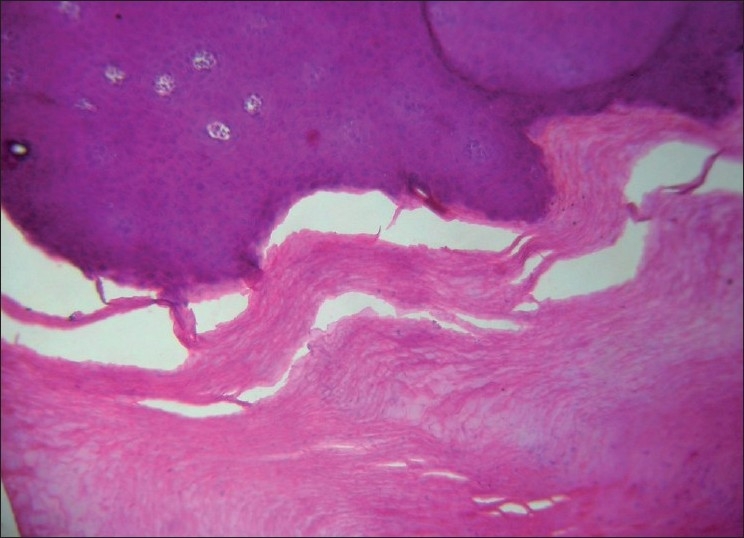
Histologic examination of palmar skin biopsy (H and E, ×10)

**Figure 6:**
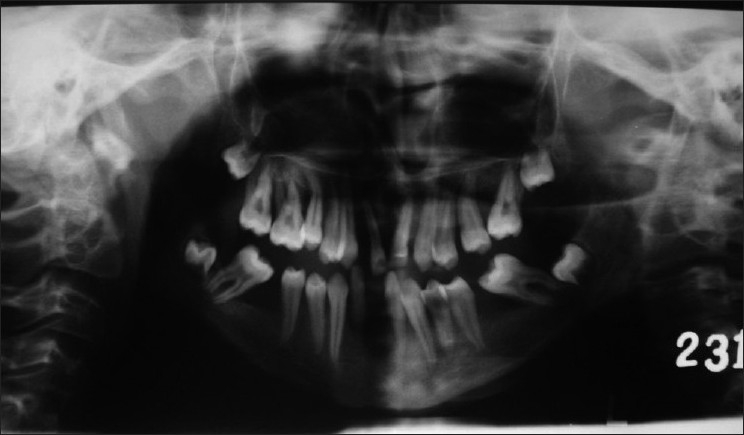
Panoramic radiograph. Note severe alveolar bone loss in relation to the permanent teeth

**Figure 7:**
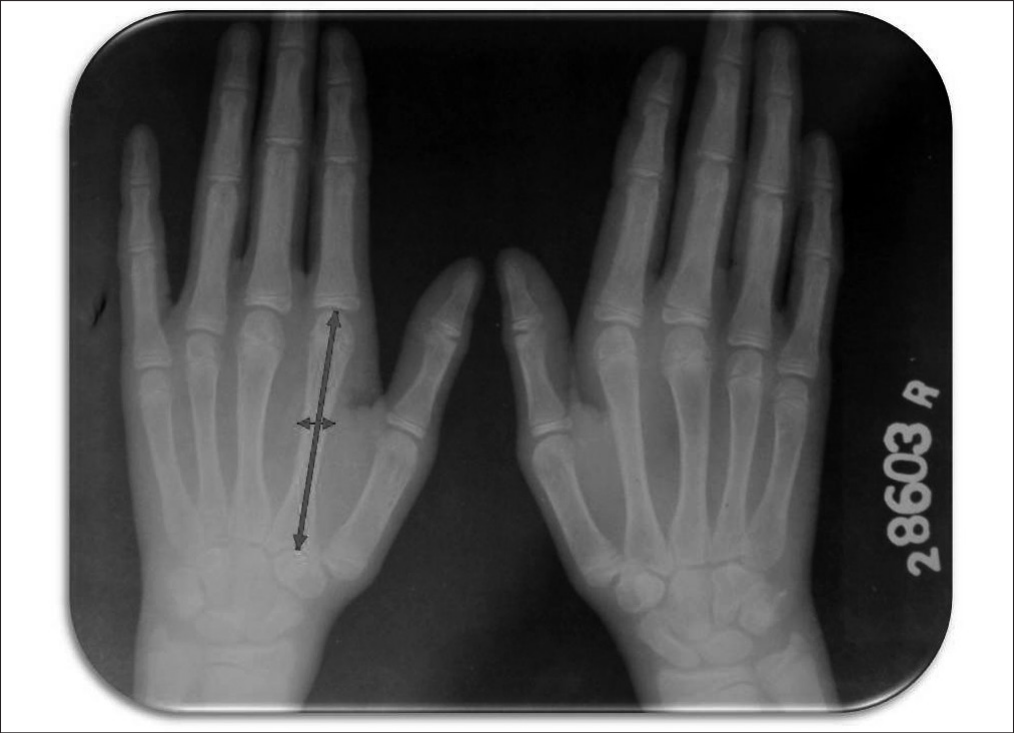
X-ray of hands. Note elongation of the metacarpals

**Figure 8:**
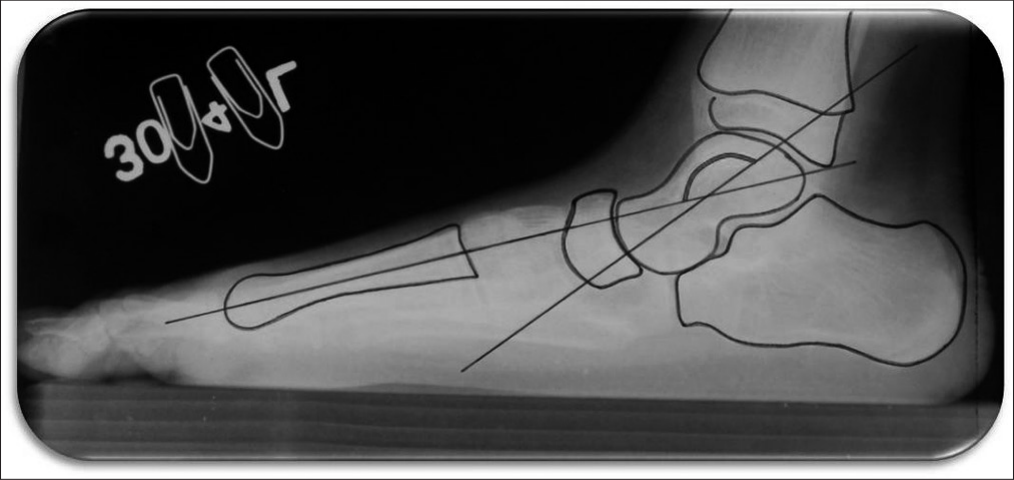
Lateral projection of feet. The line of the first metatarsal makes an angle instead of coinciding with the midtalar line

Superficial palmar biopsy revealed marked hyperkeratosis with focal parakeratosis and acanthotic epidermis [Figure 5]. X-ray of skull and CT scan of head showed no abnormality. OPG [Figure 6] of the patient showed severe alveolar bone loss in relation to the existing permanent teeth. X-ray of hands revealed that the metacarpal index was 9.3, implying elongation of the metacarpals[4] [Figure 7]. In lateral projection of feet [Figure 8], the line of the first metatarsal made an angle (instead of coinciding) with the midtalar line revealing that the proband had flat feet.[5]

### Sibling

The proband’s four-year-old brother also had complaints of bleeding from gums and mobility of deciduous teeth. His intraoral and cutaneous examination revealed similar findings.

Based on the patient’s history and clinical radiographic findings, the diagnosis of HMS was made.

Proband’s treatment consisted of phase I therapy with detailed oral hygiene instructions. Chlorhexidine digluconate mouth rinse (0.2%) was prescribed. Teeth with poor prognosis were extracted. For the first month, patient was kept on weekly recalls, with motivation of patient and reinforcement of oral hygiene. The patient was referred to the department of dermatology, for the treatment of skin lesions. The patient was given one mg/kg/day Acitretin for first three months. With seeming clinical improvement, the dose of Acitretin was reduced to 0.5 mg/kg/day.

## DISCUSSION

Haim-Munk was first described in four siblings from Cochin, India, by Haim and Munk.[6] It is an extremely rare autosomal recessive disorder characterized by congenital palmoplantar keratosis, severe early onset periodontitis, onychogryphosis, arachnodactyly and acro-osteolysis. Puliyel and Sridharan[7] were the first to describe flat feet in four cases of HMS. The estimated occurrence given by Gorlin *et al*,[1] of PLS, of which HMS is an extremely rare variant, is considered to be one to four persons per million. There appears to be no variance by gender.

The reported case resembled with PLS, but with general examination various skeletal manifestations came into highlight, suggesting it being a case of HMS. Hart *et al*,[8] distinguished HMS as a separate disorder owing to the presence of onychogryphosis, arachnodactyly, acro-osteolysis and pes planus. Although only arachnodactyly and pes planus were found in this case, still they can easily distinguish it from being a case of HMS. The criteria for arachnodactyly was given by Parish[4] who suggested a dividing line between normal and abnormal at three standard deviation level of 8.4 in men and 9.2 in women. The proband had a metacarpal index of 9.3. Further demarking this case as HMS was the absence of dural calcification, considered as a third component in PLS by Gorlin *et al*.[1]

Parental consanguinity is characteristic of both disorders, coinciding with the reported case.[9] Haneke[3] suggested that some patients have an increased susceptibility to infections, which may reflect a more deleterious effect of specific Cathepsin C mutation. The proband did have a history of repeated skin infections.

The etiologic factor reported by Hart *et al*,[9] was the missense mutations affecting both the alleles of Cathepsin C gene, located on chromosome 11q14.1-q143. The cathepsin C gene is expressed in epithelial regions such as palms, soles, knees and keratinized oral gingiva.

A typical pattern of periodontitis as reported in HMS was noticed in the proband with deciduous teeth appearing at the normal time but shedding by age of four. The permanent teeth then erupted normally followed by severe gingival inflammation.

PLS and HMS are difficult to differentiate unless the skeletal manifestations are taken into consideration. The importance of differentiating the two syndromes lies in the approach for treatment. The periodontium in HMS is thought to be less severely affected than in PLS,[9] therefore early diagnosis can aid in early intervention to retain teeth for longer; as reported in the proband, majority of the permanent teeth were in functional condition at age 12. Early identification of flat feet (seen in HMS), in childhood can help in its prevention/correction at early stages.

In conclusion, a patient with periodontitis, palmoplantar keratosis, arachnodactyly and pes planus is described here. Our findings suggest it to be a case of HMS due to the presence of arachnodactyly and pes planus which are absent in PLS.

## References

[1] Gorlin RJ, Sedano H, Anderson VE (1964). The syndrome of palmar-plantar hyperkeratosis and premature periodontal destruction of the teeth. J Pediatr.

[2] Papillon MM, Lefèvre P (1924). Two cases of symmetrical, familial (Meleda’s malady) palmar and plantar keratosis of brother and sister: Coexistence in two cases with serious dental changes (in French). Bull Soc Fr Dermatol Syphiligr.

[3] Haneke E (1979). The Papillon-Lefèvre syndrome: Keratosis palmoplantaris with periodontopathy: Report of a case and review of the cases in the literature. Hum Genet.

[4] Parish JG (1966). Radiographic measurements of the skeletal structure of the normal hand. Br J Radiol.

[5] Davis LA, Hatt WS (1955). Congenital abnormalities of the feet. Radiology.

[6] Haim S, Munk J (1965). Keratosis palmo-plantaris congenita, with periodontosis, arachnodactyly and peculiar deformity of the terminal phalanges. Br J Dermatol.

[7] Puliyel JM, Iyer KS (1986). A syndrome of keratosis palmoplantaris congenital, pes planus, onychogryphosis, periodontosis, arachnodactyly and a peculiar acro-osteolysis. Br J Dermatol.

[8] Hart TC, Stabholz A, Meyle J, Shapira L, Van Dyke TE, Cutler CW (1997). Genetic studies of syndromes with severe periodontitis and palmoplantar hyperkeratosis. J Periodontal Res.

[9] Hart TC, Hart PS, Michalec MD, Zhang Y, Firatli E, Van Dyke TE (2000). Haim-Munk syndrome and Papillon-Lefèvre syndrome are allelic mutations in cathepsin C. J Med Genet.

